# Electromembrane Extraction for Metal and Metalloid Analysis: Species-Dependent Transport, Coordinated Method Design, and Evidence-Based Translation

**DOI:** 10.3390/membranes16100349

**Published:** 2026-10-20

**Authors:** Yi Zheng, Yan Xing, Fen Wang, Hongliang Wang, Fengqing Han, Cong Li, Ying Ren, Guanglin Wang, Bin Lin, Shuang Zhou

**Affiliations:** 1 Key Laboratory of Food Safety Risk Monitoring for Health Commission of Hubei Province, Huanggang Municipal Center for Disease Control and Prevention, Huanggang 438000, China; zy19960916@163.com (Y.Z.);; 2 Hubei Provincial Hospital of Traditional Chinese & Western Medicine, Hubei Provincial Clinical Research Center for Pneumoconiosis and Poisoning, Wuhan 430015, China

**Keywords:** electromembrane extraction, metal ion, metalloid, elemental speciation, supported liquid membrane, sample preparation, matrix-specific validation

## Abstract

Electromembrane extraction (EME) applies an electric field and a supported liquid membrane to transfer charged analytes into a small acceptor phase. For metal and metalloid analysis, the field contribution is coupled to aqueous speciation, interfacial reactions, membrane composition, and acceptor-side trapping. This critical narrative review evaluates direct analytical EME evidence and distinguishes it from removal, recovery, and materials studies. The analysis links species-dependent transport and coordinated method design to the strength of evidence for different measurement claims. Available studies primarily support multication determination and Cr(III)/Cr(VI) differentiation in water, together with targeted As(V), As(III), Hg(II), and total-mercury workflows. Broader applicability to food and biological matrices remains insufficiently established. Reported enrichment or selective response alone does not demonstrate preservation of native species, matrix robustness, or universal multielement performance. Reliable translation therefore requires claim-matched validation in authentic matrices, control of species conversion, analyte-specific performance assessment, mass-balance verification, reproducible operating windows, and transparent reporting. This framework delineates the demonstrated capabilities and current evidence boundaries of analytical EME while identifying priorities for more reproducible and transferable separation and sample-preparation workflows.

## 1. Introduction

Metal and metalloid analytes are analytically demanding targets because the meaning of a measured concentration can depend on oxidation state, coordination environment, mobility, and matrix-dependent bioavailability. Mercury, cadmium, lead, chromium, arsenic, copper, nickel, cobalt, silver, and zinc may occur as hydrated cations, oxyanions, labile complexes, or forms generated during sample preparation [1,2,3,4,5,6,7]. Sample preparation should therefore transfer a defined target from a complex matrix into a detector-compatible solution while controlling contamination, adsorption, interconversion, matrix suppression, and ionic interference. For speciation analysis in particular, signal enhancement accompanied by uncharacterized changes in the original species can be analytically misleading.

Instrumental methods can provide high elemental sensitivity, but matrix simplification, preconcentration, or medium conversion is often needed before measurement. Conventional liquid extraction and diffusion-driven liquid-phase microextraction (LPME) are governed primarily by partitioning, pH, complexation equilibria, and phase-volume relationships [8,9,10,11,12,13,14,15,16,17,18,19,20,21,22]. Hollow-fiber LPME is a useful microextraction configuration because a porous fiber can retain a liquid membrane and a small acceptor volume, but transport remains predominantly diffusive and partition-driven. For inorganic ions with high hydration energies and multiple aqueous forms, successful transfer consequently depends on controlled complexation or carrier chemistry. Electromembrane extraction (EME) complements these approaches by adding an electric field that directs charged species across the membrane phase.

The analytical value of EME lies in coupling directional electromigration with control of interfacial chemistry. An electrical potential is applied between donor and acceptor phases separated by a supported liquid membrane or an equivalent membrane phase [23,24,25,26]. The observed transfer nevertheless reflects electromigration, diffusion, partitioning, interfacial complexation, and acceptor-side trapping rather than electric-field direction alone. For metal and metalloid analysis, these processes must be configured to transfer the intended ionic or complexed form into the acceptor without compromising its analytical identity. From a separation-science perspective, the relevant endpoint is therefore not membrane transfer alone but reproducible isolation of a defined target from the intended matrix with a chemically and quantitatively defensible measurement claim.

Existing reviews have examined general EME principles, device formats, environmental applications, functional materials, and liquid-membrane strategies for heavy-metal removal [27,28,29,30,31,32,33]. More recent reviews have focused on modified and hyphenated EME for food analysis [34] and on the broader development of EME as a sustainable sample-preparation platform [35]. These contributions provide valuable mechanistic, technological, and application context, but they do not specifically evaluate how the evidence for metal and metalloid EME supports different analytical claims.

Literature for this critical narrative review was identified through PubMed searches for “electromembrane extraction”, “electro-membrane extraction”, or “electro membrane extraction”, supplemented by Crossref, publisher records, and citation tracking. Searches also covered metals and metalloids, transport, carrier chemistry, speciation, validation, and alternative preparation. Titles, abstracts, and available full texts were screened. Quantitative primary studies of metal/metalloid determination by electrically driven membrane extraction were treated as direct evidence; materials-only and non-analytical removal/recovery studies were excluded from this category. Broader EME studies, reviews, guidance, and non-electrical extraction/speciation studies provided context.

## 2. Transport Mechanism in Analytical Electromembrane Extraction

EME transfers charged analytes between donor and acceptor phases separated by a supported liquid membrane (SLM) under an applied electric field [36]. For metal and metalloid analytes, field direction alone is insufficient; the target must be present in an extractable charged or complexed form and undergo controlled interfacial transfer consistent with the intended measurement claim. Figure 1 illustrates the donor–SLM–acceptor arrangement and electrode polarity for cathode-directed cation transfer.

### 2.1. EME Configurations and Polarity

Analytical EME can be implemented in several device configurations, all of which establish a donor–SLM–acceptor pathway under an applied electric field. The configurations differ in how the membrane phase and acceptor are arranged, but they rely on the same general principle of electrically assisted transfer across the SLM [37,38]. Hollow-fiber (HF) EME accounts for a substantial share of the direct metal ion application evidence summarized in Section 4 [39,40,41,42,43,44,45,46,47,48,49,50,51]. In this format, an organic liquid is immobilized within the pores of a porous fiber, while a small acceptor volume is retained in the lumen. The fiber therefore provides a confined donor–SLM–acceptor pathway for metal ion transfer. For example, Kubáň et al. used a porous polypropylene hollow fiber with an inner diameter of 1200 µm, a wall thickness of 300 µm, and a length of 3 cm, providing an approximately 30 µL lumen volume. It was impregnated for 5 s with 1-octanol containing 0.5% (*v*/*v*) bis(2-ethylhexyl)phosphonic acid [39]. The porous support retains the liquid phase and influences its effective diffusion path. Flat-membrane and miniaturized planar formats retain the same donor–SLM–acceptor principle while arranging the membrane and acceptor differently. Flat polypropylene supports, gel-based systems, in-tube gels, paper-based devices, multichannel chips, free-liquid-membrane microsystems, and alternative support materials are geometrical or support variants rather than distinct extraction principles [52,53,54,55,56,57,58,59,60]. The effects of geometry and phase-volume selection for method optimization are considered in Section 3.4.

Within any configuration, polarity links the applied field to ionic charge. For cationic transfer, the donor is connected to the positive electrode and the acceptor to the negative electrode; for anionic transfer, the electrode assignment is reversed when an extractable anionic form is present [61,62]. Polarity establishes the direction of electromigration but does not alone ensure selective extraction, because pH, complexation, and redox state determine which charged species are available for transfer. Thus, device configuration establishes the transfer pathway, polarity determines migration direction, and species chemistry determines whether the target can enter the extraction process.

### 2.2. Interfacial Transfer and Species Selectivity

Once device configuration establishes the transfer pathway and polarity determines the direction of electromigration, interfacial transfer determines whether a metal species can enter and leave the SLM. Metal ion EME involves coupled electromigration, diffusion, partitioning, and chemical reactions at the donor/SLM and SLM/acceptor boundaries. The Nernst–Planck framework describes migrative and diffusive fluxes, whereas Poisson–Nernst–Planck formulations also consider potential and charge distributions under defined assumptions [63,64,65]. These models provide a basis for interpreting flux, but they do not alone describe the interfacial reactions required to convert hydrated aqueous species into membrane-compatible forms and subsequently release them into the acceptor [66].

The SLM therefore acts as a selective reaction medium rather than an inert separator. Strongly hydrated metal ions generally require complexation, ion pairing, or carrier-assisted transfer to enter a water-immiscible membrane [10,11,12,13,14,15,18,19,20,21,22]. In the verified analytical EME studies summarized here, organophosphorus additives, ion-exchange carriers, or method-specific derivatization were used according to target charge and chemistry [39,43,44,45,46,47,48,49,50,51,67,68,69,70,71,72,73,74]. At the donor/SLM interface, the target species associates with an extractant or carrier to form a membrane-compatible complex or ion pair. The resulting species crosses the membrane under the combined influence of electromigration and diffusion. At the SLM/acceptor interface, dissociation, ligand exchange, or another controlled transformation releases the analyte into the acceptor. Acceptor-side chemistry should also limit back-transfer and, where applicable, permit carrier regeneration. Figure 2 summarizes this cycle, whose complex stoichiometry and charge remain system dependent.

Within this cycle, the relative contributions of interfacial complexation, membrane transport, and acceptor-side release to the overall transfer rate can be examined through initial-rate comparisons across carrier-activity, membrane-thickness or viscosity, and acceptor-chemistry series. Separate donor/acceptor agitation tests help assess boundary-layer effects. Defined speciation, matched interfacial area, and monitoring of current, temperature, phase volumes, and membrane integrity support interpretation. Consistent kinetic evidence from complementary comparisons helps distinguish these contributions while accounting for coupled chemical and electrical effects.

Selective transport arises from chemical and electrical filters acting before, during, and after membrane transfer. Aqueous speciation determines which charged or complexed forms are available; carrier affinity and membrane composition govern interfacial uptake; local electrode reactions can modify pH and carrier protonation; polarity establishes migration direction; and acceptor-side chemistry together with detection determines whether the transported target is retained and identified [23,24,25,26,27,28,29,63,64,65,66,75,76,77,78,79]. Species-dependent transport therefore provides the mechanistic basis for analytical selectivity, but any selectivity claim should be demonstrated for the intended target, matrix, and measurement claim using appropriate controls.

Such controls can compare carrier activity and donor pH/speciation using single-target and mixed-competitor solutions. Zero-voltage and polarity-reversal tests probe field participation, while buffer-capacity comparisons at matched initial pH assess sensitivity to electrolysis-related pH changes. Interpretation should account for buffer complexation, conductivity changes, bulk–interfacial pH differences, and field–electrode coupling. System-specific solvent-dependent voltage windows and carrier–potential interactions illustrate these dependencies [80,81]. Complementary controls and multivariable designs can assess effects and interactions within a defined experimental range [82], supporting interpretation of workflow-specific apparent selectivity.

## 3. Coordinated Method Design for Analytical Electromembrane Extraction

Section 2 established that metal ion transfer depends on the coupled effects of interfacial chemistry, species availability, and electromigration. Method development should therefore identify a coordinated operating window that preserves the intended species, maintains membrane integrity, and yields a stable analytical response. Membrane composition, aqueous-phase chemistry, electrical input, device geometry, phase volumes, and matrix properties are interdependent; a condition optimized in one configuration cannot be treated as a transferable single-variable optimum [80,83]. The following sections consider these variable groups in a practical analytical sequence, while recognizing that optimization is iterative. Figure 3 summarizes these interdependent design variables and shows how iterative optimization defines a coordinated operating window.

### 3.1. Membrane Phase and Carrier Selection

Membrane-phase design is central to analytical EME because the solvent–carrier combination defines the interfacial environment for target-species transfer. General EME literature includes water-immiscible solvents such as 2-nitrophenyl octyl ether and 1-octanol [84,85,86]. Ionic-liquid phases have been explored more broadly in total-metal and metal-speciation microextraction [87], whereas the verified metal ion EME studies summarized here predominantly use 1-octanol, often with organophosphorus additives [39,43,49,51,67]. This pattern is a practical starting point, not a universal optimum; solvent selection should match the target species, carrier chemistry, analytical matrix, and detector [88,89].

Carrier selection should be evaluated as part of the membrane-phase system rather than as an isolated additive choice. Acidic organophosphorus additives have enabled extraction of selected cations in several verified analytical workflows [39,43,51,67,90]. For anionic targets, ion-exchange or derivatization strategies may generate an extractable charged form compatible with the selected membrane and polarity [46,47,48,49,50,69,70,71,72,73,74,91]. Increasing carrier or additive concentration may promote target association but can also increase membrane viscosity and alter ionic transport, membrane resistance, and total current [89,92,93]. At higher concentrations, slower membrane transport, competing-ion uptake, or impaired acceptor-side release may offset enhanced target uptake, while changes in electrical behavior and solvent retention may affect selectivity and SLM stability. In one Ni-EME system, enrichment increased up to 0.9 M di(2-ethylhexyl)phosphoric acid (DEHPA) in kerosene and declined at the higher tested concentration, with increased viscosity proposed as a possible explanation [42]. Gel, semiliquid, and biopolymer-supported formats further illustrate that solvent retention and support properties form part of membrane-phase design [57,59,60,94]. Optimization should therefore document solvent and additive identity and concentration, support preparation, and operational evidence of membrane stability.

### 3.2. Donor and Acceptor Chemistry

Donor and acceptor chemistry determines whether the target is present in an extractable donor-phase form and can be released and retained after transfer. Donor pH, ligands, and competing constituents should maintain a soluble species compatible with the selected membrane-phase system while limiting hydrolysis, precipitation, and competing complexation [75]. Acceptor composition must promote analyte release, minimize back-transfer, and remain compatible with the intended measurement. Acidic acceptors have been used in several verified cation-transfer methods, including Hg(II) workflows [39,43,44,51,67]. However, no single acceptor composition can be generalized across elements, oxidation states, carriers, and detectors.

Within this donor–acceptor chemical framework, species stability and electrode polarity should be evaluated together. The selected electrode assignment should drive the prevailing charged form toward the acceptor without causing unacceptable interconversion or interfacial disturbance [23,24,25,26,61,62]. Buffers and complexants can alter charge state and coordination environment, whereas derivatization reagents may additionally affect species stability, transfer behavior, and detector response [14,18,19,47,48,49,50,72,73,74,75,95,96,97,98]. Optimization should therefore assess species stability and detector compatibility using separate and mixed-species standards subjected to the same reagent sequence, holding periods, electrical conditions, and detection procedure as samples. Paired pre-/post-extraction spikes help distinguish preparation and transfer effects from acceptor/detector response. For oxidation-state or native-species claims, species-resolved controls should also quantify conversion and cross-response across the complete workflow.

### 3.3. Mass Transfer Operating Conditions

After the membrane-phase and aqueous-phase system has been defined to support target-species transfer, electrical conditions provide the driving force for metal ion transport. Applied voltage must be interpreted together with electrode spacing, solution conductivity, and membrane resistance [99,100]. For geometry comparisons, a nominal field can be expressed as *E_nom_* = *V_app_/L_eff_*, where *V_app_* is the applied voltage, and *L_eff_* is the explicitly defined effective electrode separation. This descriptor differs from the local SLM field because voltage is distributed across the aqueous phases, membrane, interfaces, and electrodes. Reported conditions across hollow-fiber, flat-membrane, and on-chip methods indicate that electrical settings are configuration-dependent and should not be transferred without validation. Current should be monitored alongside voltage because it provides an operational indication of solution conductivity, electrode behavior, and membrane integrity [76,77]. Where the effective wetted membrane area *A_eff_* is defined, geometric current density is *j_geom_(t) = I(t)/A_eff_*, with *I(t)* representing total cell current rather than analyte-specific current. The area definition should specify fiber diameter and wetted length for hollow fibers, or wetted overlap/cross-sectional area for planar/gel formats. These descriptors should be calculated only when the corresponding geometry and current records are available. Excessive current can promote electrolysis, gas formation, Joule heating, or interfacial disturbance, thereby compromising recovery or reproducibility [78,79]. Temperature should be controlled or at least monitored, and deliberate temperature control should be distinguished from uncontrolled electrical heating. A stable electrical–thermal window provides the basis for selecting the remaining operating variables.

Once these conditions have been established, extraction time and hydrodynamics determine how rapidly transfer approaches a stable analytical endpoint. A time–response profile should extend beyond the candidate endpoint and track donor and acceptor amounts alongside current, pH, temperature, phase volumes, and membrane condition, rather than identify only the largest acceptor signal [101,102,103,104]. An acceptor plateau accompanied by donor depletion under stable conditions supports a depletion-limited endpoint. A subsequent acceptor decline associated with pH changes may indicate weakened trapping or back-transfer, whereas current instability, heating, or solvent loss suggests operational disturbance. Acceptor-chemistry comparisons and membrane-stability checks help distinguish these explanations [76,77,78,79]. Agitation, electrode rotation, or controlled flow can reduce aqueous boundary-layer resistance [105,106], but the selected intensity should be evaluated together with SLM integrity and the vessel or channel geometry. Extraction time and hydrodynamics should therefore be selected jointly, with the candidate endpoint assessed for stability. For example, in a seven-cation HF-EME study, extraction efficiency peaked at about 5 min for most analytes; longer extraction reduced Cu and Pb extraction, while stirring above 500 rpm provided little additional benefit [39]. These study-specific findings illustrate the need to evaluate both variables without implying a transferable optimum.

### 3.4. Geometry and Phase Volumes

The electrical and hydrodynamic operating window described in Section 3.3 is realized within a specific device architecture. Device geometry and phase volumes establish the spatial and volumetric boundary conditions for analytical EME, including diffusion-path length, interfacial area, solvent inventory, acceptor accessibility, and donor-to-acceptor mass balance. Hollow fibers, flat membranes, gel formats, and multichannel devices therefore require separate optimization even when their membrane chemistry is similar [107,108,109,110]. A small acceptor volume can increase enrichment potential, but enrichment should be demonstrated from the complete donor-to-acceptor mass balance rather than inferred from volume alone, particularly when transfer is incomplete or back-transfer occurs. More complete elemental accounting can be expressed as *M_accounted_* = *M_D_* + *M_SLM_* + *M_A_* + *M_res_* + *M_withdrawn_*, representing elemental analyte mass in residual donor solution, membrane liquid, acceptor, surface-/solid-associated residues, and permanently withdrawn aliquots, respectively. Membrane-liquid analyte and support/device residues should be recovered and measured separately using validated procedures, with surface-associated material and precipitates distinguished to avoid overlapping counts. Closure (%) is 100*M_accounted_*/*M*_0_, where *M*_0_ is the initial donor elemental mass, with the unaccounted difference, process blanks, recovered phase volumes, and uncertainty reported.

Since membrane phase and carrier selection, donor and acceptor chemistry, electrical conditions and temperature control, extraction time and hydrodynamics, and device geometry and phase volumes interact, an operating window is better defined through multivariable experimental designs than through isolated one-factor tests. Response surface methodology can be useful where the experimental design space preserves target-species integrity and membrane stability [82]. Table 1 organizes these method-design variable groups according to their roles, primary analytical checks, and the information needed for transparent interpretation of optimization choices in analytical EME, with representative study-specific numerical examples and supporting references. Once an internally consistent operating window has been established, its separation performance and analytical utility should be evaluated through target-specific validation in the intended matrix, as discussed in Section 4 and Section 5.

## 4. Analytical Applications: Measurement Claims and Strength of Evidence

An EME workflow becomes analytically meaningful only when the transport process is linked to a clearly defined measurement claim. The literature considered here addresses two fundamentally different tasks: determination of one or more elemental targets after controlled transfer, and differentiation of predefined ionic or oxidation-state forms. These tasks do not require the same level of evidence. Elemental and multication workflows require target-specific calibration, recovery, precision, interference, and matrix assessment, whereas speciation-oriented workflows additionally require evidence showing how sample preparation, extraction, derivatization, and detection affect the identity and distribution of the measured species. The applications are therefore evaluated primarily by analytical task, with matrix context treated as a determinant of evidentiary strength rather than as an independent indication of method maturity.

The available studies span model solutions, spiked environmental waters, authentic water samples, and a small number of prepared food-derived samples. These categories are not analytically equivalent. Model solutions establish transport feasibility under controlled conditions; spiked matrices test recovery in the presence of selected matrix components; and authentic samples provide stronger evidence of performance under native ionic composition, complexation, and sample-specific interferences. Prepared or digested samples support only the operationally defined elemental measurement produced by that preparation sequence. This evidence hierarchy is used below to distinguish demonstrated analytical capability from broader claims that remain unsupported. Figure 4 summarizes the measurement-claim categories discussed below and shows how matrix context shapes the strength of evidence.

### 4.1. Elemental and Multication Determination: Breadth, Miniaturization, and Matrix Transfer

Environmental waters provide the broadest direct evidence for elemental and multication EME. Hollow-fiber (HF) EME coupled to capillary electrophoresis with capacitively coupled contactless conductivity detection (CE–C^4^D) enabled multication extraction and determination in standards and water samples [39,41]. A comparative study transferred a cation-extraction concept from HF to flat-membrane EME and examined Ag^+^, Co^2+^, Cu^2+^, Cd^2+^, and Zn^2+^ before detection by atomic absorption spectrometry (AAS) [67]. Early work also demonstrated Pb^2+^ extraction from selected biological fluids [40]. Additional single-target studies addressed uranium [68], copper [111,112], zinc [113], nickel [42], thorium [114], and silver [81] in water or saline-water matrices. Collectively, these reports show that several cationic targets can be accommodated within EME-based analytical formats, but they do not establish a universal multielement method. A defensible multielement claim requires element-specific calibration, recovery, precision, interference, and matrix evidence rather than a pooled device-level response.

Miniaturized and parallel platforms extend the integration and readout capabilities of this application area but do not remove the need for target-specific validation. A three-channel on-chip system enabled parallel determination of Cr(VI), Ni(II), and Cu(II) in water samples, while related on-chip and hollow-fiber workflows provided routes for parallel metal analysis and targeted Hg(II) determination [43,44,69,115]. These studies support the compatibility of EME with smartphone-assisted colorimetry, electrochemical detection, and miniaturized analytical platforms. Nevertheless, simultaneous handling within a shared device does not demonstrate equivalent analytical performance for all targets. Calibration behavior, recovery, precision, interferences, and matrix effects should be established separately for each analyte, and results obtained for free ions in relatively simple waters should not be extrapolated to saline, particulate-containing, or ligand-rich samples without additional validation.

Evidence beyond water matrices remains preliminary and is more appropriately interpreted as limited matrix transfer than as a mature food-analysis application area. The multication HF-EME–CE–C^4^D method was applied to powdered-milk samples after dissolution or dilution in aqueous medium, whereas a separate EME–microvolume ultraviolet–visible spectrophotometry (UV–Vis) procedure was applied to total mercury determination in water and fish samples and validated against cold-vapor AAS [39,51]. These studies demonstrate feasibility under the stated preparation and calibration procedures. They do not establish broad transferability across food categories, and they should not be interpreted as evidence of native metal coordination or mercury-species preservation. The sample-treatment boundary, procedural blanks, contamination control, calibration strategy, and matrix-specific recovery are therefore integral to the analytical claim.

### 4.2. Speciation-Oriented EME: Chromium as the Benchmark and Arsenic as a Targeted Extension

Speciation-oriented EME imposes a higher analytical burden because selectivity in the final signal can arise either from differential transport of the original species or from controlled conversion and selective reaction during the workflow. Chromium currently provides direct evidence for species-resolved or operationally differentiated analytical EME. A dual-HF-EME method followed by high-performance liquid chromatography (HPLC) used oppositely directed electrokinetic transport to separate and preconcentrate Cr(III) and Cr(VI), enabling the two oxidation states to be quantified through distinct extraction pathways rather than estimating one solely by difference [45]. This constitutes comparatively direct evidence when the two pathways are independently characterized and interconversion is controlled across the complete procedure.

Other chromium workflows rely more explicitly on selective reaction chemistry. In EME coupled to electrothermal atomic absorption spectrometry (ETAAS), 1,5-diphenylcarbazide (DPC) was used to reduce Cr(VI) and form an extractable Cr(III)–DPC complex, whereas the Cr(III) initially present was determined after oxidation [47]. This is operational differentiation through controlled redox conversion, rather than unchanged Cr(VI) transport. Paper-based, dual-gel, and in-tube gel EME formats integrated extraction, controlled conversion or color development, and detection [72,73,74]. Targeted single-analyte Cr(VI) extractions have also been reported in EME or electroenhanced microextraction formats [46,48,70,71]. These approaches can improve sensitivity, miniaturization, or portability, but a selective response after derivatization does not by itself demonstrate preservation of the donor-side oxidation-state distribution. The relevant analytical unit is the complete sequence of preparation, conversion, extraction, reaction, and detection; separate and mixed-species standards, quantitative assessment of intended conversion and unintended interconversion, and matrix-matched verification are therefore needed to support estimation of the initial Cr(III)/Cr(VI) distribution from the operationally defined measurements.

For other oxyanion-forming elements, direct evidence is narrower. Targeted As(V) determination by EME–UV–Vis and As(III) determination by EME–square-wave anodic stripping voltammetry (SWASV) have been reported in water samples [49,50]. These separate methods support determination of selected arsenic oxidation states under their stated conditions, but neither establishes comprehensive arsenic speciation or preservation of the complete native species distribution. Demonstrating simultaneous preservation and determination requires mixed-species experiments in the same donor matrix, species-resolved measurements before and after the complete workflow, and quantification of each species’ recovery and interconversion, supported by independent confirmation [116,117,118]. The distinction among selective determination of one oxidation state, operational differentiation of two fractions, and comprehensive species-resolved analysis should therefore remain explicit. Table 2 presents a representative, non-exhaustive selection of primary analytical EME studies chosen to span the measurement claims, target groups, matrices, and configurations discussed in Section 4.1 and Section 4.2; for each study, it summarizes the reported conditions, available quantitative results, and evidence boundary.

## 5. Evidence Gaps and Priorities for Analytical Translation

Section 2, Section 3 and Section 4 show that analytical translation depends on alignment among the target species, coordinated operating window, matrix context, and measurement claim. The main gap is therefore not a lack of device formats, but insufficient evidence that a complete EME workflow measures a defined target reproducibly beyond the conditions under which it was developed. Future work should prioritize claim-matched validation, species-integrity control, and comparable reporting rather than signal enhancement alone.

### 5.1. From Transfer Feasibility to Matrix-Defined Validation

The direct application literature is dominated by water matrices [39,40,41,42,43,44,45,46,47,48,49,50,51,67,68,69,70,71,72,73,74,81,111,113,114,115]; prepared food samples are limited to a few studies [39,51], whereas selected biological fluids have been reported in a limited Pb(II) application [40]. Broad biological-matrix transfer and clinical robustness have not been demonstrated. These categories provide different levels of evidence: standards demonstrate transport feasibility, spiked matrices assess recovery under defined conditions, and authentic samples address native ionic composition, binding, particulates, and competing ligands. The main matrix challenges depend on the intended measurand. Protein binding and endogenous ligands can limit extractable availability or compete for carriers, while lipids can alter phase contact and particulates can affect sampling homogeneity and surface retention. For native-species claims, binding and preparation-induced conversion are key concerns, whereas total-element measurements require quantitative preparation and compatible extracts. Ionic strength can affect carrier competition and electrical behavior, as illustrated by Ca^2+^/Mg^2+^-related suppression in saline-water metal EME [41,42].

Validation should therefore specify the target form, sample-treatment boundary, intended matrix, calibration strategy, concentration range, and analytical endpoint. Authentic samples should be prioritized where feasible; otherwise, matrix-matched calibration or standard addition, procedural blanks, relevant-level recovery, and comparison with an independent method should define the remaining boundary [8,22,23]. Consistent with Eurachem’s 2025 fitness-for-purpose guidance, quantitative acceptance criteria for bias and precision, reporting-limit suitability, and measurement uncertainty should be prespecified for the intended matrix and concentration range [119]. Workflow performance should then be assessed using independent complete extractions, with measurement uncertainty estimated from relevant sources of variability. As a recovery-study planning example, the AOAC 2016 Guidelines for Standard Method Performance Requirements recommend at least three concentration levels spanning the analytical range, with at least seven independent analyses at each level [120]. These are study-design recommendations, not universal EME minimums; the design should be tailored to the intended claim and matrix-specific requirements. A matrix-validated claim requires the complete workflow to meet the prespecified, application-specific criteria rather than merely demonstrate analyte transfer or spike recovery. Evidence obtained after digestion, dilution, or other preparation applies to the resulting operationally defined analyte, not necessarily to its native form.

### 5.2. Species Integrity and Target-Specific Validation

Speciation claims require explicit distinction among preservation of an original species, operational differentiation, and intentional conversion followed by measurement. Dual-pathway chromium EME provides comparatively direct Cr(III)/Cr(VI) differentiation [45]. Within EME, DPC-assisted workflows integrate redox conversion, complexation, extraction, and detection [47,72,73,74]. Related non-EME chromium speciation studies provide complementary context for the role of controlled color development and conversion [95,96,97,98]. Existing EME studies support targeted As(V) or As(III) determination [49,50] and Hg(II) or total-mercury determination [43,44,51], rather than comprehensive speciation or preservation of native mercury species.

For a native-species preservation claim, a practical minimum evidence package spans sample handling through detection and comprises four complementary test categories. Separate and mixed-species standards assess selectivity and competition, paired pre-/post-extraction spikes distinguish workflow effects from acceptor/detector response, stability and conversion tests quantify intended and unintended transformations, and an independent speciation method checks species-resolved results [116,117,118,121]. These categories define an evidence package rather than four individual measurements, with concentrations, species ratios, and replication chosen for the intended matrix and uncertainty requirements. Recovery or signal enhancement alone cannot establish species integrity.

Multielement claims likewise require analyte-specific calibration, recovery, precision, detection capability, interference, and matrix evidence [39,67,69,115]. Mixed-analyte experiments should assess competition for carrier and matrix-dependent changes in conductivity or transport. The appropriate evidentiary unit is the target–matrix–workflow combination, not the shared device; otherwise, strong performance for one analyte may obscure weaker performance for another.

### 5.3. Reproducible Operating Windows and Comparable Reporting

Reproducibility should be demonstrated across an operating window rather than at a single optimum. Reports should document the coupled variables summarized in Table 1, including membrane preparation, phase chemistry and volumes, electrode arrangement, voltage and current behavior, temperature, time, hydrodynamics, geometry, and sample preparation [60,76,77,78,79,80,82,83,99,100,101,102,103,104,106,107,108,109,110]. Current and temperature monitoring are particularly important because electrolysis, Joule heating, local pH change, solvent loss, or membrane displacement may increase signal while reducing stability [76,77,78,79]. Robustness testing should examine conditions around the selected window. Precision studies should distinguish short-term repeatability, within-laboratory intermediate precision, and interlaboratory reproducibility. Drawing on Eurachem guidance for validation planning [119], a balanced experimental design should include independent complete extractions across days, independently prepared membrane batches, and separately assembled devices. Nested or mixed-effects analysis can estimate the corresponding variance components where appropriate to the experimental structure.

Cross-study comparison requires explicit definitions and calculation bases for extraction recovery, relative recovery, enrichment factor, detection limit, and mass balance. Enrichment should be demonstrated from measured donor-to-acceptor transfer rather than inferred from the phase-volume ratio; work with concentrated EME solutions shows that retention within the SLM may limit complete transfer, reinforcing the need for mass-balance checks [122]. Analytical translation should proceed in a logical sequence that begins with reproducible operation, advances through target-specific validation in authentic intended matrices and demonstration of species preservation or controlled conversion, and culminates in successful transfer across matrices, formats, or laboratories. Interlaboratory evaluation should use a common written protocol and blinded, homogeneous and stable representative matrix materials, with each laboratory independently preparing membranes and assembling devices. ISO 5725-2:2025 can guide collaborative study design and estimation of repeatability and interlaboratory reproducibility, while protocol deviations should be documented to aid interpretation of the observed variability [123]. This sequence favors depth of evidence over proliferation of configurations and keeps future claims within defensible analytical boundaries.

### 5.4. Comparison of EME with Alternative Sample-Preparation Techniques

The validation requirements outlined above also inform the choice between EME and alternative sample-preparation techniques. EME offers electrically adjustable, directional transfer into a small acceptor phase, combining preconcentration with matrix clean-up for compatible targets. Its operating window depends on target charge, membrane chemistry, conductivity, electrolysis, and membrane stability [76,77,78,79]. In contrast, three-phase hollow-fiber liquid-phase microextraction (HF-LPME) uses a similar donor–membrane–acceptor arrangement without electrical operation. It offers low solvent consumption and avoids electrolysis, although transfer depends on diffusion, partitioning, complexation, and acceptor trapping, which can constrain extraction kinetics [8,12]. Conventional dispersive liquid–liquid microextraction (DLLME) achieves rapid phase contact through dispersed extraction droplets. Compared with EME’s spatially separated donor and acceptor phases, DLLME requires subsequent collection of the extraction phase. Metal applications commonly rely on hydrophobic complex formation, with ligand competition and disperser effects influencing selectivity and enrichment [124].

Solid-phase extraction (SPE) shifts separation control to sorbent affinity, providing matrix clean-up, flexible sample loading, and formats suited to online coupling. Magnetic SPE (MSPE) facilitates sorbent collection, but both approaches require assessment of capacity, competitive adsorption, and elution completeness [125]. Cloud-point extraction (CPE) enriches metal chelates or ion pairs in a surfactant-rich phase and can reduce volatile organic-solvent use. Its practical limitations include temperature-dependent phase formation and surfactant compatibility with subsequent detection [126]. These alternatives provide different ways to achieve the same preparation objectives, while EME adds electrical control of membrane transfer. For species-resolved analysis, HPLC or ion chromatography (IC) coupled to inductively coupled plasma mass spectrometry (ICP-MS) can complement any of these routes through chromatographic separation and element-selective detection [5,121]. Selection should therefore match the intended target and matrix to the required clean-up and enrichment, species stability, detector compatibility, and throughput. The selected workflow should then be validated for its intended application. Table 3 summarizes the key findings, evidence gaps, and corresponding validation or selection priorities.

## 6. Conclusions

EME provides a chemically tunable approach to metal and metalloid preconcentration, but its analytical value depends on translating coordinated chemical and electrical control into a reliable measurement rather than on enrichment alone. Elemental determination, controlled conversion-based differentiation, and native-species analysis should therefore be evaluated as distinct measurement objectives. To advance these analytical applications, future work should focus on understanding species-dependent transport, extending validated workflows to more demanding matrices, and establishing when extraction preserves species identity or relies on controlled conversion. Harmonized performance definitions and reporting are needed for meaningful comparison, while robustness and reproducibility beyond the original development conditions are essential for method transfer. These priorities would strengthen the transition from proof-of-concept extraction to reliable, transferable analytical methods.

## Figures and Tables

**Figure 1:**
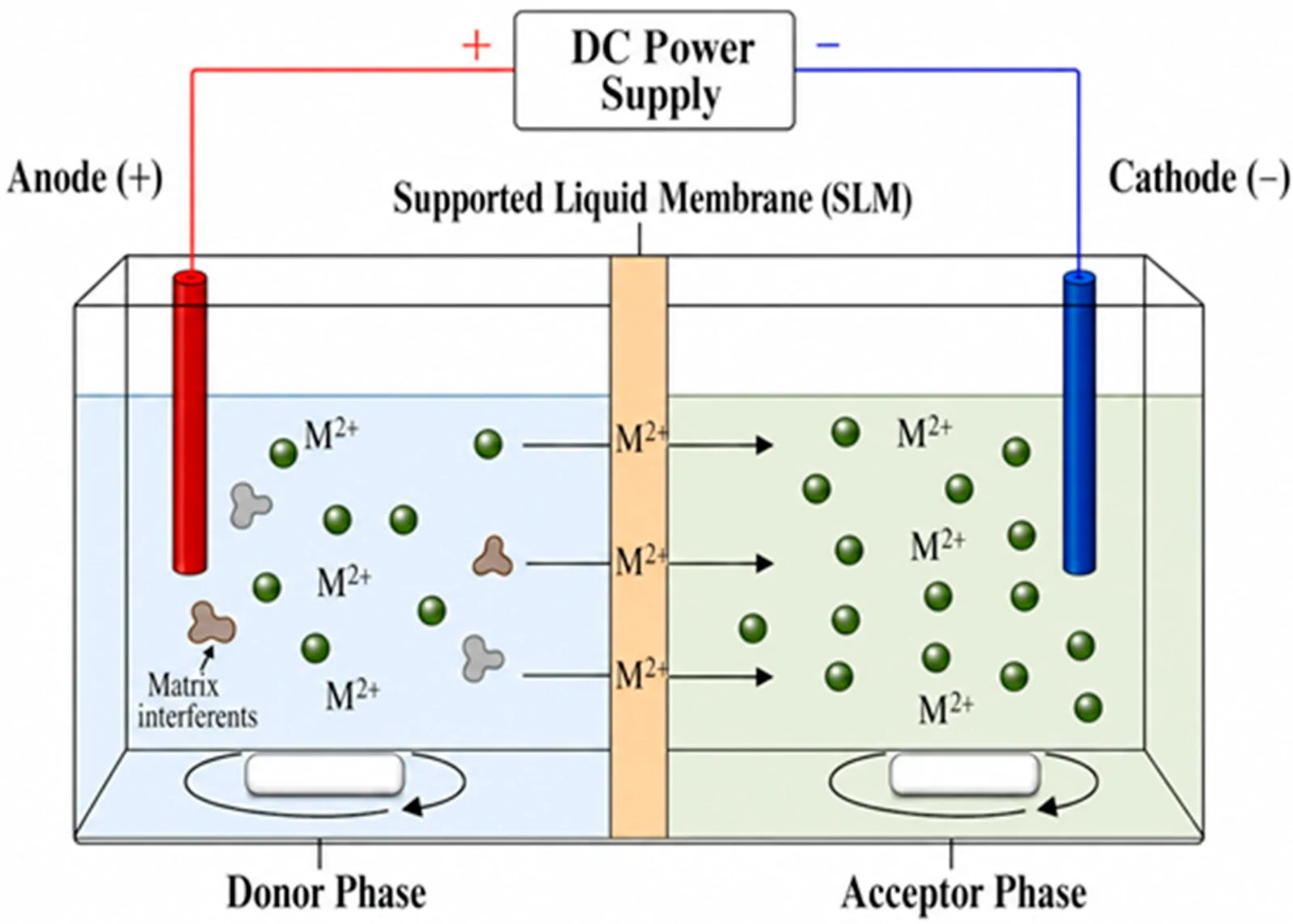
Schematic of donor–supported liquid membrane (SLM)–acceptor cell in EME for cathode-directed cation transfer. The donor and acceptor connect to the anode (+) and cathode (−), respectively; anion transfer requires reversed polarity and an extractable anionic form. M^2+^ denotes the aqueous target class, not transport of a bare hydrated ion.

**Figure 2:**
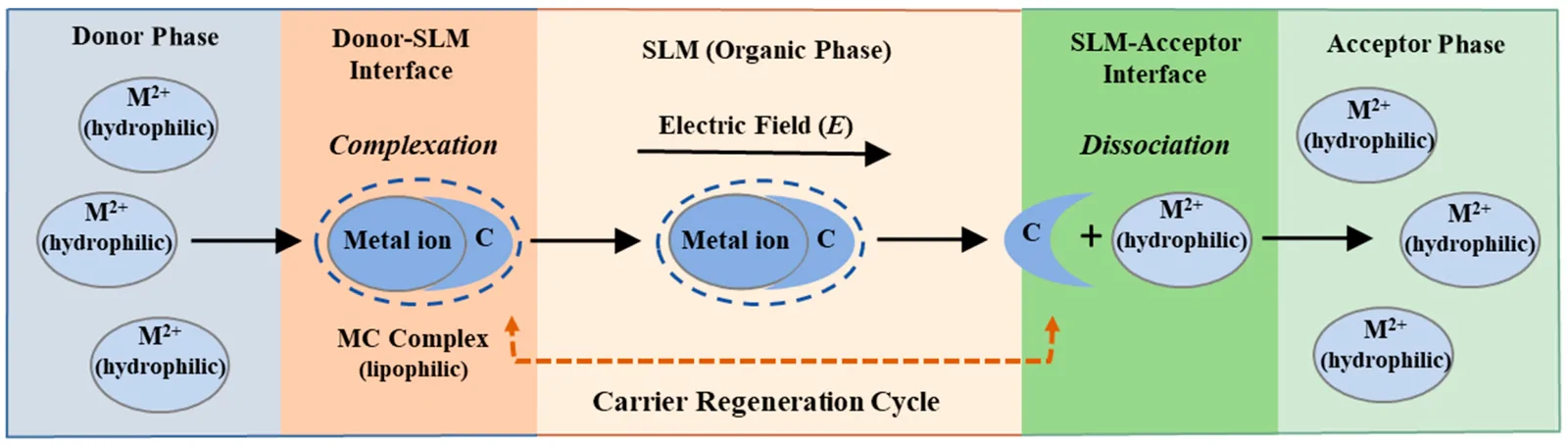
Schematic association and transfer cycle for a representative divalent metal ion (M^2+^) in a supported liquid membrane (SLM). The stoichiometry and net charge of the transported metal–carrier species, the relative contributions of electromigration, diffusion, and interfacial reactions, and the extent of carrier regeneration or recycling depend on the system.

**Figure 3:**
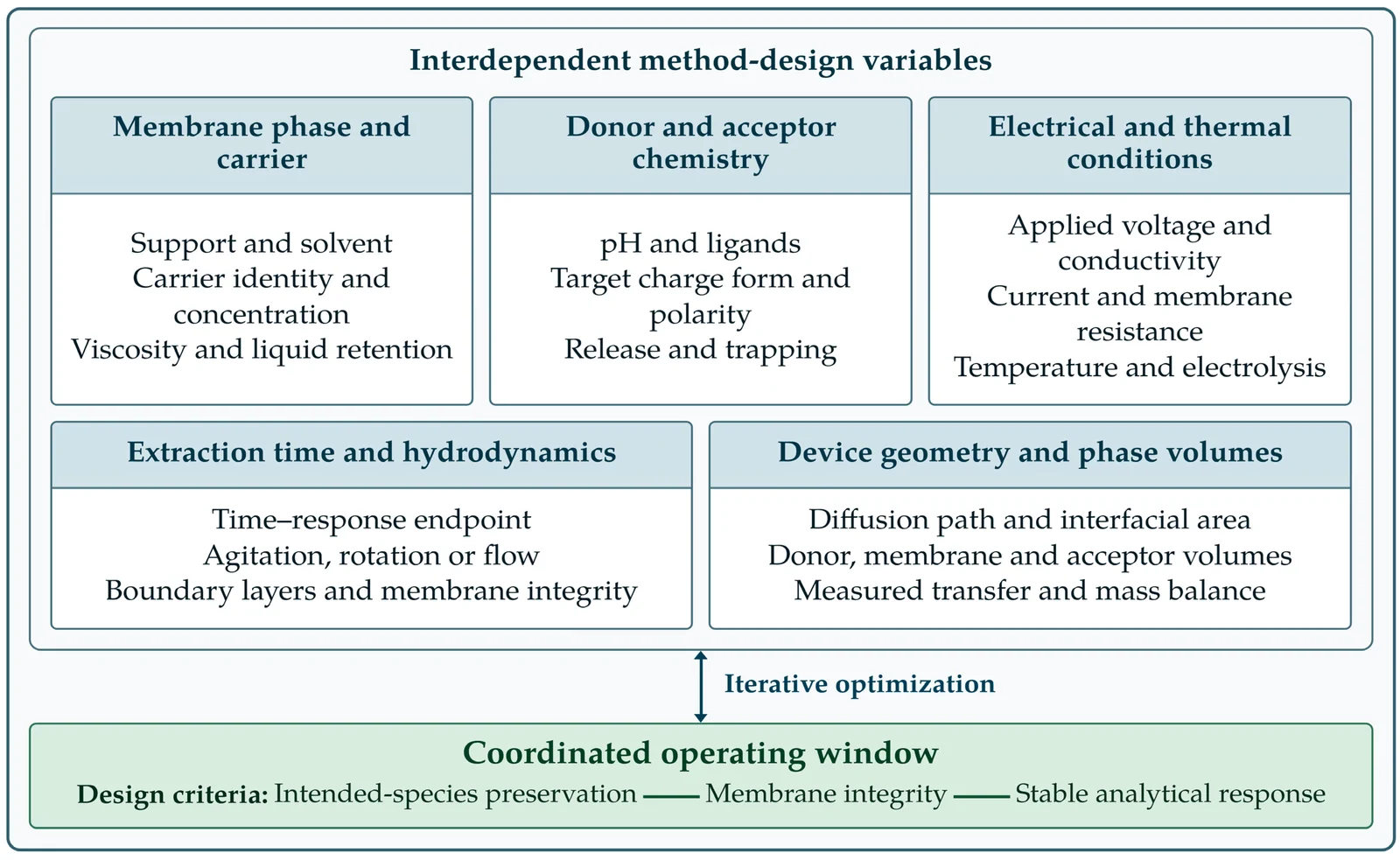
Coordinated method design for analytical EME. The interdependent method-design variables discussed in Section 3 are considered iteratively to establish a coordinated operating window. Intended-species preservation, membrane integrity, and a stable analytical response are treated as design criteria.

**Figure 4:**
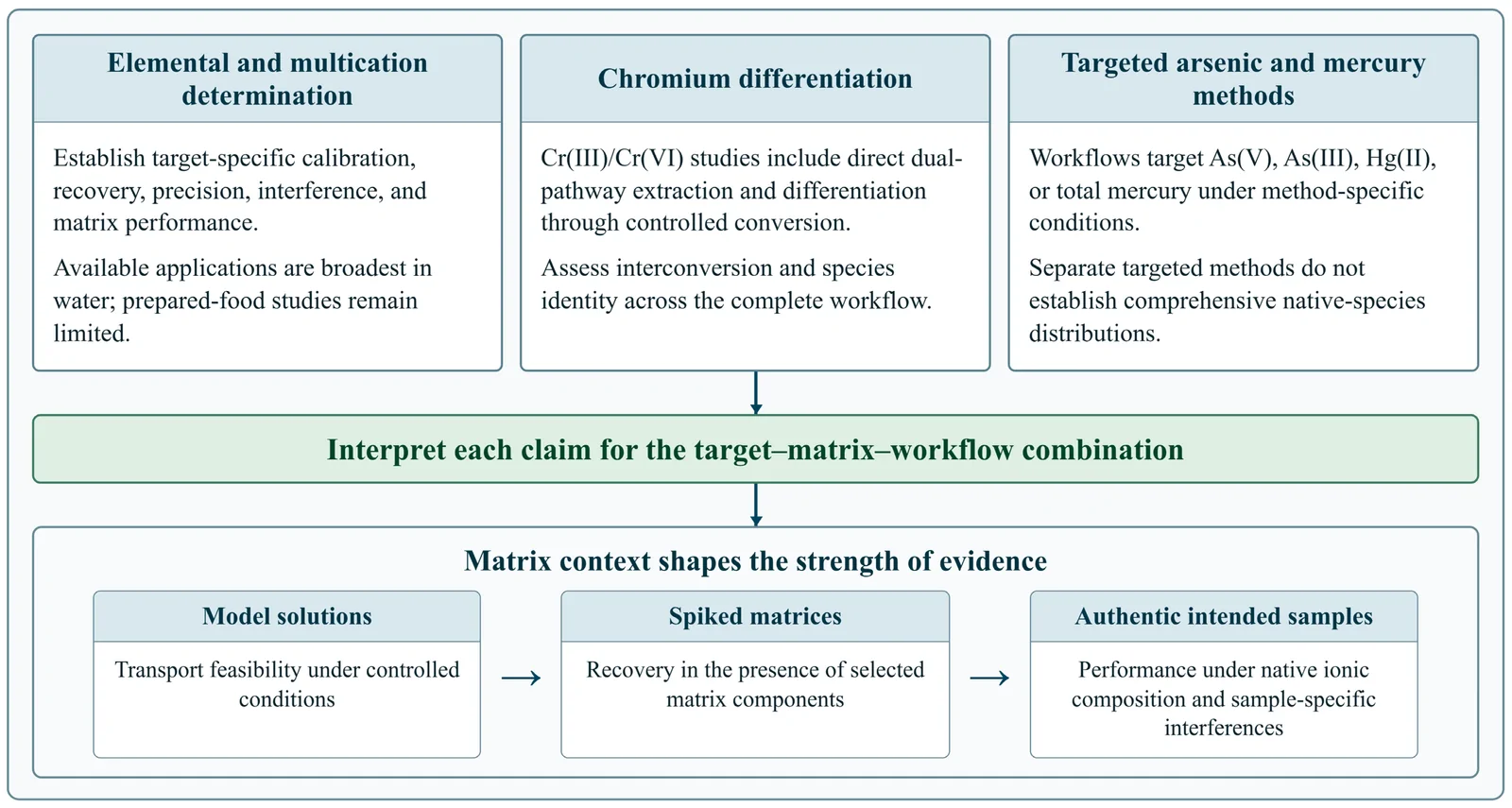
Measurement claims and evidence context in analytical EME. The upper boxes distinguish elemental and multication determination, chromium differentiation, and targeted arsenic or mercury workflows, each requiring evidence matched to the target, matrix, and complete workflow. The lower boxes indicate increasing relevance to authentic sample contexts, from model solutions through spiked matrices to authentic intended samples; the arrows do not represent required sequential steps or a numerical method-maturity score.

**Table 1:** Coordinated method-design and reporting framework for analytical electromembrane extraction (EME).

Method-Design Variable Group	Analytical Function	Key Checks During Optimization	Information Needed for Interpretation	Representative Study-Specific Quantitative Examples	Ref.
Membrane phase and carrier system	Establishes the interfacial environment for membrane entry, transport, and acceptor-side release by controlling target–membrane compatibility, carrier-mediated association, and membrane-phase mobility.	Target- and charge-form compatibility; selectivity relative to coexisting ions; carrier-concentration response; viscosity-related changes in transport and electrical behavior; acceptor-side release; solvent leakage, membrane displacement, and operational SLM stability.	Membrane solvent and carrier identity, purity, and concentration; support material, dimensions, wetted area, and pretreatment; impregnation procedure and solvent loading, where determined; preparation-to-use interval; observations or measurements used to assess membrane stability across independent preparations.	Low-electroendosmosis agarose containing 5% (*w*/*v*) dextrin was used for gel EME of Cr(III)/Cr(VI)	[53]
Donor and acceptor chemistry	Defines the extractable donor-side species and promotes analyte release and retention in the acceptor while maintaining compatibility with the intended detection procedure.	Target solubility and prevailing charge form; hydrolysis, precipitation, and competing complexation; species stability or interconversion; consistency between target charge and electrode polarity; acceptor-side trapping and back-transfer; effects of buffers, ligands, masking agents, or derivatization reagents on extraction and detection.	Donor and acceptor composition, pH, volume, and ionic strength or conductivity, where relevant; concentrations of buffers, ligands, masking agents, and derivatization reagents; electrode polarity; sequence and timing of reagent addition; separate/mixed-species and pre-/post-extraction spike controls; species stability and conversion controls.	A total-mercury EME workflow used donor and acceptor pH values of 7 and 3, respectively.	[51]
Electrical and thermal operating conditions	Provide the electromigrative driving force and define the electrical and thermal operating window within which extraction proceeds without unacceptable disturbance of the membrane or aqueous phases.	Voltage–current–time behavior; current stability; effects of electrode spacing, solution conductivity, and membrane resistance; electrolysis, gas evolution, local pH change, and Joule heating; membrane disturbance or solvent loss; distinction between controlled extraction temperature and uncontrolled electrical heating.	Power-supply mode and applied voltage or current; electrode material, geometry, placement, and spacing with defined *L_eff_* and wetted area *A_eff_*; current–time profile *I(t)*; *E_nom_* = *V_app_*/*L_eff_* and *j_geom_(t)* = *I(t)*/*A_eff_* where the required records are available; temperature setpoint and/or monitored temperature profile; criteria and observations used to define electrical, thermal, and membrane stability.	A flat-membrane metal-cation EME method used an applied voltage of 60 V	[67]
Extraction time and hydrodynamics	Determine the rate of approach to a predefined analytical endpoint and regulate aqueous boundary-layer mass transfer.	Donor/acceptor amount–time profiles extending beyond the candidate endpoint; donor depletion and acceptor accumulation; onset of plateauing or decline in response; pH changes, membrane loss, and back-transfer; effects of stirring, agitation, or flow on recovery, precision, and SLM integrity.	Time range evaluated and selected extraction time; endpoint criterion; agitation or flow mode, intensity or rate, and orientation; vessel, channel, or flow-cell geometry; current, bulk pH, temperature, recovered phase volumes, and membrane condition during the time-response study; recovery and precision at the selected conditions.	An As(V) EME workflow used an extraction time of 15 min and a stirring rate of 750 rpm	[49]
Device geometry and phase volumes	Define diffusion-path length, exposed interfacial area, membrane-solvent inventory, acceptor accessibility, and the spatial and volumetric boundaries governing donor-to-acceptor transfer.	Configuration-specific effects of path length and interfacial area; phase-volume effects on recovery and enrichment; acceptor accessibility and sampling; incomplete transfer and back-transfer; donor-to-acceptor mass balance; enrichment verified from measured transfer rather than inferred from phase-volume ratio alone.	Device format and construction; membrane thickness or fiber dimensions and exposed area; donor, membrane, and acceptor volumes; relevant channel or vessel dimensions; sampling procedure and withdrawn aliquots; equations and definitions used for extraction recovery, relative recovery, enrichment factor, and mass balance; separate donor, membrane-liquid, acceptor, and surface/solid measurements; closure, unaccounted mass, blanks, and uncertainty.	A seven-cation HF-EME setup used a 3-cm Accurel polypropylene 300/1200 fiber (1200 µm inner diameter, 300 µm wall thickness, approximately 30 µL lumen), with 3.5 mL donor and 20 µL acceptor.	[39]

Note: EME, electromembrane extraction; SLM, supported liquid membrane. The listed items represent minimum reporting and interpretive fields for coordinated EME method development. No universal optimum is assigned because the appropriate operating window is configuration-, target-, carrier-, matrix-, and detector-dependent.

**Table 2:** Representative analytical electromembrane extraction (EME) applications classified by measurement claim, matrix context, and evidence boundary.

Analytical Task and Target(s)	Matrix Context and Evidence Level	EME Configuration and Readout	Representative Reported Conditions	Key Analytical Performance	Supported Analytical Claim and Evidence Boundary	Ref.
Multication determination: Mn^2+^, Cd^2+^, Pb^2+^, Zn^2+^, Co^2+^, Cu^2+^, and Ni^2+^	Standards, tap water, and prepared powdered-milk samples; standard-addition calibration for real samples	HF-EME–CE–C^4^D	1-octanol + 0.5% *v*/*v* DEHP; 75 V; 5 min; 750 rpm; 100 mM acetic acid acceptor	ER 15–42%; RSD 6.6–11.1%; LOD 25–200 nM	Supports rapid multication pretreatment and determination in the tested aqueous and prepared samples. Unequal recoveries and matrix sensitivity preclude a universal multielement claim.	[39]
Configuration transfer: Ag^+^, Co^2+^, Cu^2+^, Cd^2+^, and Zn^2+^	Model aqueous media, real aqueous samples, and diluted nanosilver suspension	Flat-membrane versus HF-EME–AAS	1-octanol + 0.5% *v*/*v* DEHP + 0.5% *v*/*v* TEHP; 60 V; 10 min; donor pH 10; acceptor pH 1.2	More than 71.2% of selected free ions were extracted from the initial aqueous samples under optimized conditions	Supports transfer of a cation-extraction concept from hollow fiber to flat membrane and application to selected aqueous samples. It does not establish configuration equivalence or performance in undiluted complex matrices.	[67]
Parallel on-chip determination: Cr(VI), Ni(II), and Cu(II)	Water samples; analyte-specific channels and relative-recovery assessment	Three-channel on-chip EME–smartphone RGB colorimetry	Target-specific SLM and donor/acceptor conditions; the reported platform used separated extraction and chromogenic channels	Linear ranges: 0.05–1.5 mg L^−1^ for Cr(VI), 1.0–70.0 mg L^−1^ for Ni(II), and 0.5–30.0 mg L^−1^ for Cu(II); intra-/interday RSD <6.9%/<7.8%; RR 85.0–116.4%	Supports parallel, smartphone-assisted determination in the tested waters. Shared-chip operation does not substitute for analyte-specific validation and does not alone establish field robustness.	[69]
Targeted Hg(II) determination	Model solutions and spiked tap and river waters	HF-EME–SWASV using a AuNP-modified glassy-carbon electrode	1-octanol + 2.0% *v*/*v* DEHP; 60 V; 12 min; 700 rpm; 15 µL of 100 mM HCl acceptor	Linear range 0.2–10 µg L^−1^; EF 102–108; RR 89–97%; RSD 7.5–8.7%; LOD 0.01 µg L^−1^	Supports sensitive Hg(II) determination in the tested water matrices. The narrow range and spiked-matrix evidence do not establish total-mercury or native mercury-species analysis.	[43]
Total mercury determination	Water and fish samples after the study-specific preparation procedure; comparison with cold-vapor AAS	HF-EME–microvolume UV–Vis	1-octanol + 2% *v*/*v* DEHP; 70 V; 10 min; 700 rpm; donor pH 7; acceptor pH 3	Linear ranges 2.3–950.0 µg L^−1^ in water and 40–9500 µg kg^−1^ in fish; LOD 0.7 µg L^−1^ and 12 µg kg^−1^, respectively; CV < 6%	Supports total mercury determination in the tested water and prepared fish samples. It does not support preservation or differentiation of native mercury species.	[51]
Direct differentiation of Cr(III) and Cr(VI)	Standards and authentic tap, mineral, and river waters	Dual-HF-EME followed by HPLC–UV after complexation	Oppositely directed extraction into two hollow fibers; study-specific conditions for the two pathways	Linear range 20–500 µg L^−1^; recovery 31.1–47.2%; preconcentration factor 21.8–33; RSD 9.8–13.7%; LOD < 5.4 µg L^−1^	Supports separate extraction and quantification of Cr(III) and Cr(VI) in the tested waters. Initial oxidation-state preservation requires full-workflow conversion controls. Moderate recovery and water-only validation limit broader matrix claims.	[45]
Operational Cr(III)/Cr(VI) speciation through controlled conversion	Real water samples	HF-EME–ETAAS with 1,5-diphenylcarbazide-mediated chemistry	Cr(VI) reduction and Cr(III)–DPC complex extraction; initial Cr(III) determined after oxidation	Linear range 0.05–5 ng mL^−1^; EF 110; recovery 66%; RSD 3.7–12.2%; LOD 0.02 ng mL^−1^	Supports sensitive operational differentiation of Cr oxidation states in water when the conversion sequence is controlled. It is not evidence of unchanged transport of the original donor-side species.	[47]
Portable and miniaturized Cr(III)/Cr(VI) differentiation	Tested water samples, including authentic and spiked waters	Paper-based single/dual EME, dual-gel EME, and in-tube gel EME coupled to µPAD colorimetry	Method-specific 1-octanol/DEHP SLM or agarose-gel pathways, DPC color development, and controlled conversion steps	Paper-based workflow: LOD 1.0 µg L^−1^ for Cr(III) and 0.7 µg L^−1^ for Cr(VI); dual-gel workflow: ER 83.3% and 58.8%, with LOD 3.0 and 2.0 µg L^−1^, respectively; in-tube gel workflow: ER 84% for Cr(III) and 72% for Cr(VI), with an LOD of 7.0 µg L^−1^ for both species	Supports low-cost, miniaturized chromium differentiation in the tested waters. Performance is workflow-specific, and color-selective response does not alone prove preservation of the initial oxidation-state distribution or applicability to more complex matrices.	[72,73,74]
Targeted arsenic oxidation-state determination: As(V) or As(III)	Model solutions and tested water samples	HF-EME–UV–Vis for As(V); HF-EME–SWASV using a AuNP-modified glassy-carbon electrode for As(III)	As(V): 1-octanol + 2.5% *v*/*v* DEHP, 70 V, 15 min, and 750 rpm; As(III): study-specific extraction conditions	As(V): linear range 5–300 ng mL^−1^, recovery 95–102%, RSD 3.4–7.6%, and LOD 1.5 ng mL^−1^; As(III): two linear ranges of 0.5–10 and 10–600 µg L^−1^, RSD 6.7–8.5%, and LOD 0.18 µg L^−1^	Supports separate, targeted determination of As(V) or As(III) under the respective method conditions. Neither study establishes simultaneous or comprehensive arsenic-species analysis or preservation of the complete native species distribution.	[49,50]

Note: AAS, atomic absorption spectrometry; AuNP, gold nanoparticle; CE–C^4^D, capillary electrophoresis with capacitively coupled contactless conductivity detection; CV, coefficient of variation; DEHP, bis(2-ethylhexyl) phosphate; DPC, 1,5-diphenylcarbazide; EF, enrichment factor; EME, electromembrane extraction; ER, extraction recovery; ETAAS, electrothermal atomic absorption spectrometry; HF, hollow fiber; HPLC, high-performance liquid chromatography; LOD, limit of detection; RR, relative recovery; RSD, relative standard deviation; RGB, red–green–blue; SLM, supported liquid membrane; SWASV, square-wave anodic stripping voltammetry; TEHP, tris(2-ethylhexyl) phosphate; UV–Vis, ultraviolet–visible spectrophotometry; µPAD, microfluidic paper-based analytical device. The table presents representative direct analytical studies rather than an exhaustive inventory. Numerical values and conditions are study-specific and are not transferable optima. Here, “speciation” denotes the claim supported by the complete workflow; where controlled oxidation, reduction, or derivatization is used, the result is more accurately treated as operational differentiation unless preservation of the original species distribution is independently demonstrated. This evidence hierarchy provides the basis for Section 5.

**Table 3:** Key findings, evidence gaps, and validation priorities for the analytical translation of EME methods for metals and metalloids.

Topic	Key Findings/Available Evidence	Remaining Limitation	Validation or Selection Priority	Supporting Sources
Matrix-defined validation	Evidence is concentrated in waters, with limited prepared-food and biological-fluid applications. Ni EF was 180 ± 17 during optimization and 27 ± 2 in synthetic seawater.	Optimization or matrix spiking alone does not establish general matrix transfer.	Use authentic intended matrices, full-process validation, suitable reference materials or independent methods, and prespecified performance requirements.	[39,40,41,42,51]
Species integrity	Cr workflows support direct or conversion-based differentiation. As(III)/As(V) and Hg workflows are targeted and method specific.	Selective recovery or detector response does not demonstrate native-species preservation or comprehensive speciation.	Use separate/mixed-species standards, pre-/post-extraction spikes, stability/conversion tests, and independent species-resolved confirmation when required by the claim.	[45,47,48,49,50,72,73,74,116,117,118,121]
Transfer accounting	Enrichment depends on measured transfer and recovered phase volumes. Analyte retention in the membrane can limit transfer.	A donor/acceptor phase-volume ratio alone cannot establish the mass transferred, and an acceptor-side result alone cannot demonstrate closure of the elemental mass balance.	Define EF/recovery and account separately for donor, membrane liquid, acceptor, residues, and withdrawn aliquots where relevant.	[42,76,77,78,79,122]
Reproducibility and transfer	A Ni study reported intraday RSD 5.1% and interday RSD 15%. Preparation, device, and laboratory effects require separate evaluation.	Short-term repeatability is not interchangeable with intermediate precision or interlaboratory reproducibility.	Monitor current, temperature, and membrane integrity across the operating window. Include independent complete extractions across days, membrane batches, and devices, then evaluate interlaboratory reproducibility under a shared protocol.	[42,119,123]
Alternative preparation methods	EME adds electrical control. HF-LPME, DLLME, SPE/MSPE, and CPE offer passive membrane, droplet, sorbent, and surfactant-phase routes.	Heterogeneous analytes, matrices, volumes, and detectors prevent a universal superiority ranking.	Select the route to match target form, matrix, clean-up/enrichment, species stability, detection, and throughput.	[8,12,124,125,126]

Note: Observed evidence and future requirements are distinct. Source-specific metrics do not define universal EME thresholds.

## Data Availability

The original contributions presented in this study are included in the article. Further inquiries can be directed to the corresponding authors.

## Abbreviations

The following abbreviations are used in this manuscript:

AAS	Atomic absorption spectrometry
AuNP	Gold nanoparticle
CE–C^4^D	Capillary electrophoresis with capacitively coupled contactless conductivity detection
CPE	Cloud-point extraction
CV	Coefficient of variation
DEHP	Bis(2-ethylhexyl) phosphate
DEHPA	Di(2-ethylhexyl)phosphoric acid
DLLME	Dispersive liquid–liquid microextraction
DPC	1,5-diphenylcarbazide
EF	Enrichment factor
EME	Electromembrane extraction
ER	Extraction recovery
ETAAS	Electrothermal atomic absorption spectrometry
HF-LPME	Hollow-fiber liquid-phase microextraction
HPLC	High-performance liquid chromatography
IC	Ion chromatography
ICP-MS	Inductively coupled plasma mass spectrometry
LOD	Limit of detection
LPME	Liquid-phase microextraction
MSPE	Magnetic solid-phase extraction
RGB	Red–green–blue
RR	Relative recovery
RSD	Relative standard deviation
SLM	Supported liquid membrane
SPE	Solid-phase extraction
SWASV	Square-wave anodic stripping voltammetry
TEHP	Tris(2-ethylhexyl) phosphate
UV–Vis	Ultraviolet–visible spectrophotometry
µPAD	Microfluidic paper-based analytical device
